# ‘Maternal request’ caesarean sections and medical necessity

**DOI:** 10.1177/14777509231183365

**Published:** 2023-06-26

**Authors:** Rebecca CH Brown, Andrea Mulligan

**Affiliations:** 1 Oxford Uehiro Centre for Practical Ethics, Oxford, UK; 2 School of Law, 8809 Trinity College, Dublin 2, Ireland

**Keywords:** caesarean section, medical necessity, informed consent < clinical ethics, autonomy, childbirth, decision making

## Abstract

Currently, many women who are expecting to give birth have no option but to attempt vaginal delivery, since access to elective planned caesarean sections (PCS) in the absence of what is deemed to constitute ‘clinical need’ is variable. In this paper, we argue that PCS should be routinely offered to women who are expecting to give birth, and that the risks and benefits of PCS as compared with planned vaginal delivery should be discussed with them. Currently, discussions of elective PCS arise in the context of what are called ‘Maternal Request Caesarean Sections’ (MRCS) and there is a good deal of support for the position that women who request PCS without clinical indication should be provided with them. Our argument goes further than support for acceding to requests for MRCS: we submit that healthcare practitioners caring for women with uncomplicated pregnancies have a positive duty to inform them of the option of PCS as opposed to assuming vaginal delivery as a default, and to provide (or arrange for the provision of) PCS if that is the woman's preferred manner of delivery.

## Introduction

Currently, many women who are expecting to give birth have no option but to attempt vaginal delivery, since access to elective planned caesarean sections (PCS) in the absence of what is deemed to constitute ‘clinical need’ is variable. In this paper, we argue that PCS should be routinely offered to women who are expecting to give birth, and that the risks and benefits of PCS as compared with planned vaginal delivery should be discussed with them. Currently, discussions of elective PCS arise in the context of what are called ‘Maternal Request Caesarean Sections’ (MRCS) and there is a good deal of support for the position that women who request PCS without clinical indication should be provided with them. Our argument goes further than support for acceding to requests for MRCS: we submit that healthcare practitioners caring for women with uncomplicated pregnancies have a positive duty to inform them of the option of PCS as opposed to assuming vaginal delivery as a default, and to provide (or arrange for the provision of) PCS if that is the woman's preferred manner of delivery.

## Access and attitudes to maternal request caesarean section

Access to MRCS varies between and within countries. In many countries CS is seen as an intervention to be reserved for particularly difficult or high-risk births, and planned vaginal delivery is the presumed and recommended default mode of delivery for the majority of women. There has, however, been some movement to recognise that women might, in some circumstances, nonetheless prefer to deliver via CS rather than vaginally, even where medical opinion is that this is not necessary. In the United Kingdom, for instance, official guidelines now recommend that women who request caesarean births in the absence of medical indication should be provided with one. Such a decision should come after a discussion of their reasons for requesting a CS and after ensuring that they are informed of the associated risks of different modes of birth.^1^ These recommendations are not, however, mandatory. Local commissioners and healthcare providers are not required to provide MRCS and, according to a survey by Birthrights,^2^ whilst about a quarter of National Health Service trusts follow the NICE guidelines, nearly half have policies that are only partially consistent with the guidelines, and 15% explicitly do not offer MRCS. Individual obstetricians may also ‘conscientiously’ object to performing MRCS. Survey data from England and Wales in 2001 suggested just under 30% of obstetricians would refuse a request for MRCS.^3^

We don’t have space to survey different national policies or approaches to MRCS provision. However, a number of countries in Europe, North America, and Australia are roughly similar to the UK insofar as MRCS is permitted or advised in the absence of medical indication where a woman has been made fully aware of the harms and benefits of different modes of birth and still prefers MRCS (see e.g.^4,5^ The International Federation of Gynaecology and Obstetrics (FIGO) published a position paper in 2018 called ‘How to stop the caesarean section epidemic’ which describes “an alarming increase in caesarean section” worldwide, and insisting “The rise in CSs has to be stopped.” ^6^ Despite clear scepticism about CS where no medical indication is present (they describe this as a ‘medically unreasonable’ treatment option) they do not, however, recommend against providing women with MRCS. Online guidance states “If, after… efforts to inform the patient's request [for a non-clinically indicated mode of birth] have been completed and she is therefore able to make an informed and voluntary request, it is ethically permissible to implement her request.”^7^ Norway is an example of a European country where MRCS is not provided for in guidelines: According to Eide and Bærøe^8^ the Norwegian health system guidelines make CS dependent on medical indication, where ‘medical indication’ is understood to mean the anticipated benefit of providing a CS is higher than proceeding with vaginal delivery.^a^ The view of the World Health Organisation appears to be that healthcare systems should aim to reduce rates of CS performed without clinical indication, and it characterises these CS as “unnecessary”.^9^

As indicated by the situation in the UK, however, it would be a mistake to assume that official guidelines that are sympathetic to MRCS will result in the reliable provision of MRCS. It is often unclear the extent to which women who wish to receive a CS without medical indication are able to do so. It is also worth noting that we are not aware of any national or similar healthcare guidelines which recommend spontaneously offering PCS without it being requested. That is, guidelines, at most, recommend providing MRCS when a woman has independently made this request, and only after an in depth consideration of her reasons for the request and full discussion of the harms and benefits of CS versus planned vaginal delivery. Whilst early discussions of a woman's birthing options may be recommended (e.g., opting for delivery in a midwife-led unit versus consultant-led care in a hospital), this does not seem to include the consideration of PCS unless there is reason to think one is clinically indicated. It might also be the case that, whilst officially tolerated or even supported, MRCS is actively discouraged by healthcare professionals ‘on the ground’.^3,10^

## Planned caesarean should be (routinely) offered

### MRCS is medically reasonable

The starting point for our argument is that the evidence demonstrates that elective PCS is medically reasonable for low-risk pregnancies. We note that for PCS to be medically reasonable is much less demanding than for it to be medically necessary. The latter requires, at minimum, that PCS be associated with greater clinical benefits and fewer clinical harms than the available alternatives. Medical reasonableness, as we use the term, only requires that PCS achieve some threshold level of clinical harm/benefit,^b^ and that it not be *vastly* worse than the alternative (vaginal delivery or, more specifically, trial of labour).

We will not provide a full discussion of the relative health harms and benefits of PCS versus trial of labour here. This is a fairly contentious area, partly because the availability and quality of evidence is limited. We are concerned exclusively with planned CS in uncomplicated pregnancies, rather than with complicated pregnancies or emergency CS. However, a particular challenge arises from the difficulties in distinguishing between outcomes associated with different kinds of CS: planned CS which is performed because the pregnancy is high risk and vaginal delivery is deemed unacceptably risky; planned CS in low-risk pregnancies where there is no medical indication for CS but where the woman has a (non-medical) preference for CS; emergency CS where labour has commenced with a plan for vaginal delivery but where labour does not progress as hoped and a decision is taken to instead perform a CS. There are differences both in the procedures involved and the cohort undergoing these procedures. Yet analyses of the outcomes in CS versus vaginal delivery are generally poor at accounting for these differences, and so expected outcomes for MRCS are often confounded by outcomes for other kinds of CS.

That said, guidelines such as those produced by NICE which seek to correct for such confounds and evaluate the effects of MRCS specifically find that the outcomes are comparable. There are a number of outcomes that are thought to be more likely with a CS birth (including maternal and neonatal death) and a number more likely with a vaginal delivery (e.g., urinary incontinence and severe pain).^1^ Some of these differences in outcomes are small, some are large. The guidelines also acknowledge that there is conflicting evidence for some outcomes, and that there are likely to be relevant differences in outcomes for specific individuals that are not included in the tables provided.

The crucial point is that planned vaginal delivery, as well as CS, presents significant risks of negative outcomes. For instance, according to the NICE guidelines, just under half of women who deliver vaginally experience urinary incontinence more than 1 year after birth; about 15% experience faecal incontinence. Delivering vaginally is also, often, exceptionally painful: the median pain score for women giving birth was 8/10 (with 10 being the most severe pain). The NHS advises that up to 9 in 10 first-time mothers who deliver vaginally will suffer a tear, graze or episiotomy, and that one in 7 deliveries in England involves an episiotomy.^11^

If we accept the NICE guidance, then PCS versus trial of labour looks like a ‘preference-sensitive decision’ in a robust sense. That is, a decision where there is no clear medical case for the superiority of one mode of birth over the other. The higher risks of certain outcomes in CS might be compensated for by the lower risks of other outcomes, in a way that only the individual birthing woman can decide which is more important for her. For instance, some might prefer a shorter duration of extreme pain over a longer duration of more moderate pain. The (dis)value of such options is inherently subjective. If mode of birth were truly considered a preference-sensitive decision in this way, then it would be clear that both PCS and planned vaginal delivery should be presented to women with low-risk pregnancies as equally (medically) suitable childbirth options.

Whilst we think this is plausible, we don’t wish to defend this strong claim. Instead of claiming that the harm/benefit profiles of MRCS and vaginal delivery make this a robustly preference-sensitive decision (where medical opinion finds them essentially quantitatively indistinct, although qualitatively different), we claim that they are both *medically reasonable* options. That is, MRCS (and vaginal delivery) involve expected health harms and benefits that women could reasonably accept, given their situation, and neither is significantly (medically) superior in a way that makes the other an unreasonable choice.

As we noted, the evidence here is often of poor quality and the interpretation is disputed. It is hard to make a case for robust preference-sensitivity. But it is not hard to make a case for medical reasonableness. The evidence is sufficiently indicative of there not being unacceptable harms associated with MRCS, even though some dispute whether the purported benefits outweigh the harms.^7,12,13^ Yet in other areas of medicine, patients are not required to choose what is considered by their care team to be the ‘medically best’ option. They are, instead, presented with the reasonable alternatives, and generally recommended that which is considered medically most appropriate, and then given the opportunity to consent or refuse consent to those options. Childbirth should be no different. Current approaches to childbirth present trial of labour as the default course of action, with the alternative of CS only presented to the woman if she requests it, or if there is some particular clinical indication. This is despite the fact that vaginal delivery poses an unavoidable risk of physical injury.

Finally, a further reason for thinking that MRCS is a medically reasonable birthing option is the likelihood of attempted vaginal delivery resulting in an emergency CS. This is arguably the worst outcome: it can involve many of the downsides of vaginal delivery (e.g., severe pain) plus those of CS (e.g., longer recovery) and few of the upsides (e.g., no opportunity to plan timing or prepare in advance). Emergency CS is a relatively common outcome of planned vaginal delivery: Scottish public health guidance states that around 17% of deliveries are via emergency CS.^14^ Data for healthy women with low-risk pregnancies giving birth in England shows an emergency CS rate of around 10%.^15^ The same study found that instrumental deliveries, such as ventouse and forceps, occurred in around 6%–7% of such low risk deliveries. A good way of avoiding an emergency CS or instrumental delivery is to opt for a planned CS. This is, at the very least, something that pregnant women should be informed of and have the opportunity to consider.

### Women should get the opportunity to choose their injury

We have argued that rather than being medically necessary, PCS need only be a medically reasonable option in order to justify a requirement to offer it to women as a mode of birth option. By specifying medical reasonableness as the test, rather than medical necessity, the influence that clinical factors play in delimiting women's decisions about their mode of both is significantly reduced. We think this is appropriate having regard to the fact that vaginal birth, as well as PCS, poses an unavoidable risk of injury to the woman. Although vaginal delivery is generally presented as the default and preferred option for low-risk pregnancies, it is misleading to present planned vaginal delivery as risk free, or not to discuss the risks at all.^16^

The risks of vaginal delivery, and the injuries that a woman may sustain by reason of it, are routinely underestimated in discussions of childbirth. This is perhaps because vaginal delivery is seen as inevitable, and therefore the injuries sustained are regarded as unavoidable consequences of pregnancy. To appreciate the magnitude of the injuries associated with vaginal delivery, it is useful to examine them through the prism of “compensable damage”. This is a legal concept used in the tort of negligence to delineate damage in respect of which the courts will order a wrongdoer to pay compensation, from damage that a victim might perceive as subjectively harmful, but for which the courts will not order compensation. The classic example of a non-compensable injury is distress. While a wrongdoer might cause you grave distress, it will not be compensable unless you can satisfy exacting criteria, such as demonstrating a recognised psychiatric illness or an associated physical injury. The reproductive sphere generates many interesting controversies concerning compensable damage, such as whether parents can recover for the costs of raising a child that came into existence by reason of a negligently performed sterilisation operation^17^ or whether a severely disabled person should be able to recover damages in respect of the hardship of their own life.^18^ However, what is always regarded as entirely uncontroversial is the fact that a woman can recover for the injury associated with pregnancy and childbirth. Even in the highly technical and ideologically charged arena of financial recovery for reproductive injuries, no one disputes that pregnancy and labour are compensable personal injuries.^17,19^ We think this is a useful fact on which to reflect in the context of debates around PCS. Elective PCS is often treated with suspicion because there is a sense that women should not be entitled to voluntarily undergo a serious injury. This view fails to appreciate that all women facing childbirth are going to encounter some kind of injury. We simply believe they should be entitled to choose which kind of injury they would prefer to suffer.

Framed in terms of risk rather than injury, we say women should be forewarned of the risks of trial of labour and given the opportunity to decide whether or not they are exposed to these risks, or the set of risks associated with alternative modes of birth such as PCS. As already mentioned, it is sometimes argued that it is not in women's best interests to deliver via PCS if they are carrying a low-risk pregnancy, and thus they should not be offered PCS.^20^ The guidelines in use in Norway, which explicitly make CS dependent on clinical need, and even those in the UK and elsewhere, where MRCS is recommended *once a woman has spontaneously requested one* (and after extensive discussion) but where MRCS is not routinely offered, implicitly endorse the principle that healthcare professionals should first decide what is in the birthing woman's best interest (i.e., planned vaginal delivery in the case of low-risk pregnancies) and then only offer/make them aware of that option. This is not how disclosure of treatment alternatives ought to proceed, nor is it how disclosure of treatment options proceeds in other clinical contexts. It is not for third parties to decide on behalf of a person with capacity what is in their interests and then list information accordingly. Failure to disclose the risks and benefits of PCS as compared with planned vaginal delivery denies a woman the opportunity to make her own assessment of the choices she faces. As argued, PCS, whilst not necessarily in a woman's best (medical) interests, is a medically reasonable alternative and ought to be disclosed as such to pregnant women so as to facilitate autonomous decision-making.

Our position is consistent with legal principles of informed consent as contained in *Montgomery v Lanarkshire Health Board.*^21^ *Montgomery* is widely regarded as a seminal re-orientation of the law of informed consent towards the rights of the patient, and in particular, the patient's right to autonomy. The decision recognises the fundamental subjectivity of healthcare decision-making on the part of the patient. The Court commented:

*The relative importance attached by patients to quality as against length of life, or to physical appearance or bodily integrity as against the relief of pain, will vary from one patient to another. Countless other examples could be given of the ways in which the views or circumstances of an individual patient may affect their attitude towards a proposed form of treatment and the reasonable alternatives. The doctor cannot form an objective, “medical” view of these matters, and is therefore not in a position to take the “right” decision as a matter of clinical judgment.* [Paragraph 46]

When PCS is only made available on maternal request (and possibly not *even* made available on maternal request) this involves the substitution of an “objective, medical view” of delivery for what should be a personal decision on the part of the pregnant woman. The preference between the risks of PCS and planned vaginal delivery is highly subjective and highly personal, just as *Montgomery* contemplates.

Furthermore, *Montgomery* characterises the duty of the doctor to disclose material risks of injury as “*the counterpart of the patient's entitlement to decide whether or not to incur that risk*” (Paragraph 82). By this logic, if women are, as per the NICE guidelines, entitled to be provided with PCS on request, then there is a duty to disclose the comparative risks of PCS and planned vaginal delivery. This is a logical result of the fact that PCS is, as we argue above, a medically reasonable option. Finally, on *Montgomery,* it is worth pausing to consider the relevance of the particular facts of that case. It concerned a diabetic woman – Nadine Montgomery – who was pregnant with a large baby. She repeatedly raised her concerns about the size of the baby but was never informed of the risk of shoulder dystocia – which, at 9–10%, was substantial – nor was she offered the option of delivering via CS to avoid that risk. Her baby encountered shoulder dystocia during delivery and was later diagnosed with cerebral palsy and Erb's palsy. Nadine Montgomery underwent a highly traumatic labour, which involved a partial symphysiotomy. The truly remarkable evidence of the consultant obstetrician was that while she was aware of the risk of shoulder dystocia and the fact that CS would avoid it, she did not disclose the risk or offer CS because she did not believe CS was the better option and she believed that disclosure would make women likely to elect to undergo CS (Paragraph 13). The Supreme Court was extremely critical of this approach, commenting that it effectively meant that the patient was prevented from taking an informed decision (Paragraph 95). *Montgomery* concerned CS in the context of a high-risk pregnancy, and is therefore not on all fours with the low-risk pregnancies on which we are focused. However, the principles regarding choice and CS are pertinent. While clinicians may not believe that CS is the best clinical option, this should not absolve them of the duty to discuss it with patients and provide it if that is the patient's preference.

We have focused on the harm/benefit trade-offs for a woman expecting to give birth, and not directly discussed the different expected outcomes for the baby. This is largely for space reasons, but we expect a consideration of the baby's interests (and who gets to determine these) will not change the analysis. First, the interventions we discuss are done to the birthing woman / involve some intrusion upon her body, and thus normal procedures requiring her consent apply. The baby is not an independent person with decision-making capacity. Second, the empirical evidence regarding harm/benefits for the baby depending on mode of birth also fails to show that one mode of birth is clearly (clinically) preferable. Third, even in the case of a born child, parents typically act as surrogate decision-makers for children who lack the capacity to decide for themselves regarding medical interventions. By analogy, the mother is the person who should be responsible for deciding which mode of birth is appropriate given a concern for both her own and her baby's interests.

### Medical necessity is not a helpful concept here

The language of “necessity” pervades discussions about CS. For example, the literature routinely examines rates of “unnecessary” CS,^22,23^ and the WHO supports policies that aim to reduce “unnecessary” CS (WHO 2018). We argue that concepts such as medical necessity, clinical/medical need and clinical/medical indication have limited use as determinants for access to CS. First, it is now widely accepted that even if a CS is deemed ‘medically necessary’ for a particular woman, she must not be forced to have one. Instead, assuming she has capacity, she must be informed of the severity and risks of her situation, the likely outcomes if she receives a CS and the likely outcomes if she refuses one and/or opts for one of a number of alternative interventions. This issue has been extensively debated in the literature as well as being litigated before the courts.^24–26^ Second, as we have argued, the absence of medical necessity doesn’t indicate that a CS shouldn’t be performed (or offered).

Romanis^27^ has argued that women's reasons for requesting MRCS will, often, count as ‘clinical’ in a sense that should indicate a ‘clinical need’ for a CS. Romanis points out that the majority of requests for MRCS

are made by people with underlying health conditions they fear may be exacerbated by vaginal delivery or later emergency caesarean, and by individuals with experience of previous traumatic birth or other traumatic experience (eg, sexual assault).^27^

So it seems that ‘clinical need’ might just be too narrowly applied at present, and ought to be expanded to include a range of broader considerations that impact a person's health, taking seriously psychological health as well as physical. This still seems, however, to neglect things that matter (often deeply) to people, yet which probably should not be considered part of their ‘health’ or framed as valuable only insofar as they impact health. For instance, a preference for CS might be partly informed by a need to be able to plan when one gives birth. Such a preference might be dismissed as prizing convenience over health, but who should be the judge of what matters most to a person? Someone with limited childcare options for existing offspring, or who acts as a carer for others, for instance, might find it incredibly valuable to be able to pre-determine when they will give birth so as to ensure they are able to make alternative care arrangements. This should not be framed as a ‘health need’, but nor should it be dismissed as trivial. While expanding the definition of ‘clinical need’ may expand the practical availability of MRCS, on a principled level it does not address the fundamental problems we identify in constraining access to PCS on this basis. Nor does it address the need for a positive duty to disclose the risks and benefits of PCS versus planned vaginal delivery to women who may not be aware of them, and thus unable to access MRCS because they never ask for it. Indeed, the vulnerable women that Romanis identified might be especially in need of healthcare practitioners who actively inform them of these options.

Not only does the language of ‘medical necessity’ fail to discriminate between instances where a CS should be performed and cases where it shouldn’t, and fail to recognise the importance of non-clinical values, it reinforces the view of vaginal delivery as ‘natural’ or ‘default’, and deviations from this as inherently undesirable. Whilst vaginal delivery might be natural in some sense, the way this term is often used – to imply a normative rather than descriptive judgement – is unhelpful and inappropriate. Aside from the conceptual confusion around what counts as ‘natural’, it hardly needs stating that plenty of so-called natural occurrences are harmful or bad in some way, and it is erroneous to place value on something purely because of its apparent naturalness. There may well be reasons why women prefer to avoid interventions during childbirth, and many of these may stem from preferences for a ‘natural’ birth. There are good reasons for respecting those preferences despite qualms we might have regarding their origin or rationality. But that is no reason to reinforce the beliefs underlying preferences for naturalness and non-intervention where these beliefs are epistemically questionable. The language of necessity assumes that there are certain injuries that a birthing woman must simply endure by default, without any entitlement to a say in which injuries those are.

## Objections

### Patients have a right to refuse treatment but not to demand their preferred treatment

One might be concerned that an argument such as we provide would mean that healthcare providers become obliged to fulfill the treatment preferences, however ill-advised, of patients. The General Medical Council of the UK (GMC) guards against this in their guidance. In *Personal Beliefs and Medical Practice* they state: “The law does not require doctors to provide treatments or procedures that they have assessed as not being clinically appropriate or not of overall benefit to the patient.”^28^ This was recognised as a legal principle in *R(Burke) v General Medical Council*,^29^ a rather complicated case where the Court of Appeal ruled that medical staff could not be obliged, in advance, to provide life sustaining treatment to a terminally ill patient at some point in the future when he had lost capacity to communicate his wishes, and where the provision of such life-sustaining treatment might not be judged in his interests by his medical team. The principle has also been recognised by courts in other common law jurisdictions (eg., High Court of Ireland^30^). In general, doctors’ clinical discretion is given heavy weight in ethical and legal disputes, and it is thought that it would be undermining of *their* autonomy and professional integrity to require them to provide treatment which they do not think is clinically appropriate.

Yet, as we have already argued, 1) it is accepted that there is likely nothing clinically inappropriate about providing MRCS in low-risk pregnancies (as supported by NICE guidelines and other experts) and 2) clinical factors appear to be overweighted in this context. Given that PCS is medically reasonable and likely to be preferred by some women, where individual doctors disagree they ought to refer women under their care to doctors who are willing to provide the (reasonable) care requested. In the NICE guidance, it is acknowledged that some doctors may not wish to provide MRCS, and the guidance recommends referral to alternative care providers in such cases. We think this is an appropriate response. It is not clear that an objection to providing elective PCS is actually a conscientious objection rather than a clinical objection, but we think that useful comparison can be drawn with systems of care that protect a right to conscientious objection on the part of healthcare providers, but simultaneously impose upon those practitioners a duty to refer the patient onwards to someone that will provide the treatment. When this is regulated properly, individuals retain their right not to provide, but the healthcare system in which they operate takes responsibility for ensuring availability of services.^31,32^

The provision of PCS is not, therefore, a case where a patient is making a wild and unreasonable request for inappropriate treatment that would undermine doctors’ professional integrity.^8,33^ It is not, for example, a request for medication with no prospect of benefit, or a request to amputate a healthy limb.^34^ Pregnant women are placed in a vulnerable position where they are very likely to experience (potentially serious, potentially traumatising) injury at a future point, one which medical staff is positioned to influence. PCS ought to be one of the available birthing options for women in such a situation. This is not equivalent to saying that people have a general ‘right’ to any treatments they deem desirable, but rather that in the specific context of childbirth, ethical medical practice ought not to foreclose options on the basis of a narrow view of clinical benefit and paternalistic judgements of what is in a patient's best interest.^c^

### There is a risk that women will feel pressured to deliver via CS

As we have noted, there is widespread concern about the ‘alarming’ increase in CS rates.^6^ Countries such as Brazil, where CS accounts for more than 50% of deliveries, are sometimes pointed to as examples where the enthusiasm for CS has gone too far. There is evidence that reasons for high CS rates can include pressure from medical staff (who may benefit financially, or in terms of convenience, from performing CS) and misunderstanding of the relative harms and benefits of CS versus vaginal delivery.^35–38^

Yet concern that women are being pressured into modes of birth contrary to their interests and, perhaps, contrary to what their preferences would be where they are appropriately informed, does not seem like a good reason for limiting women's options further. As persuasively argued by Romanis,^27^ if one is concerned that women's choices are being railroaded, the solutions lie in seeking to empower those choices rather than limit them further. At this point, it is highly speculative to assume that opening up access to PCS will result in many women feeling undue pressure to choose this as the mode of birth. Indeed, we might speculate that the current situation in much of the world is one where women experience an unfortunate degree of pressure (in the form of social stigma and medical disapproval) to give birth in an ‘unmedicalised’ manner.

It might be suggested that the mere offer of PCS could be (mis)interpreted as a recommendation.^39^ This seems at least plausible. Once again, however, the appropriate solution does not seem to be one of avoiding the offer altogether and limiting choice. Instead, it recommends guarding against this interpretation by explicitly disowning it. It is important that women are engaged in conversations about the harms and benefits of different modes of childbirth and part of these conversations can include recommendations on the basis of apparent clinical benefit/harm trade-offs.

While it is clearly important to avoid a scenario whereby women feel pressured into choosing CS, it is not legitimate to oppose disclosure of PCS as an option on the grounds that women might choose it. This position is founded on the same grave error seen in *Montgomery –* a clinician whose personal opposition to CS meant that she denied her patient the information she needed to make an informed decision.

### Routinely offering PCS will result in large increases in the number of CS the health system has to perform and which it cannot afford

This is the most potentially persuasive objection to the routine offering of PCS to pregnant women. Whilst much of the discussion about why rising rates of CS focuses on the apparent health harms to women (and babies) that will result from medically unnecessary CS, an underlying concern is that healthcare systems cannot afford the additional cost. There are two separate premises that need to be established: 1. an empirical premise and 2. a normative premise. The empirical premise involves establishing that PCS is more expensive than the reasonable alternatives, and that routinely offering PCS would cause rates to increase (perhaps significantly). The normative premise is that assuming the empirical premise is established, limited healthcare resources make it unjust to spend additional resources providing PCS.

Is the empirical premise correct? First, it is not clear how CS rates would change if women were routinely offered PCS. There might be immediate changes in CS rates due to the greater availability of PCS, and longer-term changes as attitudes towards PCS change. Birthrights, a UK-based charity that campaigns for human rights in childbirth, note that enquiries about PCS are the most common reason for women to contact them via their email service.^2^ So there may be at least some unmet demand for PCS. Yet this may not result in massive increases in CS rates. While we argue that PCS is medically reasonable, we fully acknowledge that it comes with significant risks and costs that many women would wish to avoid, even if offered. There is some evidence to suggest that women would not opt for elective PCS in great numbers: a survey in Sweden found that pregnant women preferring a CS in the absence of medical indication was around 7% in first-time mothers before they gave birth, and around 10% 1 year post-partum (when considering preferences for any future children they might birth).^40^

The significance of any increase in CS rates is dependent on the resource costs of such increases. It is common to assume that CS is (significantly) more expensive than vaginal delivery. After all, it necessitates an operating theatre, a surgeon, an anaesthetist, and a longer stay in hospital after delivery. Vaginal delivery, on the other hand, can occur at home or in a midwife-led unit, and the mother and baby can be discharged hours rather than days after delivery. Yet the economic costings conducted for the NICE guidance^41^ suggest that once adverse effects such as incontinence (and the cost of treatment for this) are included in the cost-effectiveness analysis, MRCS is only marginally more costly than planned vaginal delivery. Specifically, when some of the ‘downstream’ costs of planned vaginal delivery are accounted for, the economic model puts CS as costing an extra £84 per delivery.^41^

We lack the space (not to mention expertise) to offer much more analysis of the economic costing of different modes of birth. It is apparent that this is a complicated process, and will be significantly influenced by the extent to which downstream externalities are accounted for, and methodological features of how health states are valued (NICE uses ‘quality adjusted life years’ to capture treatment benefit, but these can be contentious). It seems at least plausible, however, that PCS is not significantly more costly than planned vaginal delivery in the UK context. This means the empirical premise is not clearly established.

The normative premise assumes that, if PCS is more expensive, it would be unjust to use resources on this rather than on something else. This might be true if, for instance, providing PCS meant it became necessary to forego providing other medical treatments that are better justified. Consider a highly simplified (unrealistic) case: there is one anaesthetist available who is about to help deliver a baby via CS being performed at maternal request. However, another woman who has been attempting vaginal delivery urgently needs a CS. The second woman's life will be endangered if she does not receive a CS within the next hour. It seems clear, in such a scenario, that the second woman's need for a CS should take precedence over the first. If rationing of obstetric care in the real world looked like this – where there was frequently a direct conflict between providing elective PCS and life-saving emergency CS – then it would be unethical, in that situation, to provide elective PCS (and, indeed, to offer it to patients). Yet most healthcare systems do not operate at a point where only immediately lifesaving care can be justifiably provided. In the UK, a cost-effectiveness measure of cost per quality adjusted life year (QALY) is used to determine whether a particular intervention provides sufficient ‘bang for your buck’. As a rough approximation, NICE typically approves interventions as sufficiently cost-effective if they cost no more than £20–30,000 per QALY.^42^ Taking account of the costs and QALY losses of urinary incontinence, the NICE guidelines conclude that, although MRCS is less cost-effective than planned vaginal delivery, it should still be considered a cost-effective alternative to vaginal delivery.^41^

This is not to say that a system such as the NHS could cope if the number of PCS suddenly vastly increased. Planning and provision of the needed services would be necessary, as would an assessment of the likely impact of routinely offering PCS to pregnant women, so as to estimate the necessary changes in service provision.

## Concluding remarks

We have argued that PCS should routinely be offered to pregnant women with low-risk pregnancies. We claim that PCS is a medically reasonable option, where the clinical harm/benefit profile is not unacceptable or vastly (quantitatively) different from that of alternatives, such as planned vaginal delivery. We have argued that medical necessity fails to track the distinction between appropriate and inappropriate medical care and is thus not helpful when considering whether or not PCS should be offered and provided to pregnant women. Childbirth is very likely to be accompanied by some kind of injury, ranging from relatively minor and transient to permanent and life-altering. Women ought to be given information about the different kinds of risks they are exposed to through different modes of childbirth, and the opportunity to make an informed choice about which injuries they (and their babies) are more likely to experience.

## Notes

a. Whilst neither of the authors of the current paper speak Norwegian, google translate suggests one of the recommendations from the Norwegian guidelines is “We do not recommend caesarean section at the woman's request alone and in the absence of a medical indication”^43^

b. This will vary depending on the individual's situation. For those in unfortunate circumstances, all interventions and non-interventions alike will have poor harm/benefit profiles. Yet they still must select some option, and it is reasonable to do so.

c. There is an additional debate here about whether women – particularly once labour has commenced – have capacity to make decisions about things such as the advisability of CS (see, for instance^12^). First, we think this is just mistaken, and that there is no good reason to think that birthing women in general, lack capacity. Second, it is routine to seek informed consent from birthing women during labour, so it is clear that normal practice assumes such women have capacity, and unclear why they should be considered to lack capacity with regard to decisions about CS. Finally, almost all MRCS will be planned prior to labour, and so the threat that labour poses to capacity will be irrelevant.
